# Safety and efficacy of a suction cervical stabilizer for IUD insertion: Results from a multicenter post‐marketing study

**DOI:** 10.1002/ijgo.70718

**Published:** 2025-12-03

**Authors:** Michal Yaron, Victoria Crofts, Ana C. Marcelino, Ximena Espejo‐Arce, Elaine Garcia, Julia Barbe, Elisabeth Garcia Vilaplana, Micol Murtas, Cassia T. Juliato, Dehlia Moussaoui, Alissa Conklin, Luis Bahamondes

**Affiliations:** ^1^ Department of Woman, Child & Adolescent University Hospitals (HUG) Genève Switzerland; ^2^ Department of Obstetrics and Gynecology University of Campinas Faculty of Medical Sciences Campinas SP Brazil; ^3^ University Obstetricians‐Gynecologists Coleman Center Indianapolis Indiana USA

**Keywords:** bleeding, intrauterine device, nulliparous, pain, real‐life study, satisfaction, suction cervical stabilizer, transcervical

## Abstract

**Objective:**

To evaluate the experiences with an atraumatic device (Carevix®) for intrauterine device (IUD) placement in routine clinical practice.

**Methods:**

Our prospective, post‐marketing study was conducted in 19 centers in Europe, Brazil and the USA. IUD insertions using Carevix device were documented for completion rates, patient‐reported pain scores, cervical bleeding, and operator and patient satisfaction. Demographics, uterine position, and menstrual cycle phase were also recorded.

**Results:**

A total of 1123 IUD placements were analyzed. Most participants (819/992; 83%) were 18–40 years old; 46% (513/1123) were nulliparous and 47% (526/1123) were menstruating at the time of the procedure. The procedure was completed with Carevix alone in 82% (925/1123) of participants. Additional tenaculum use was mostly due to spontaneous vacuum loss (194/1123). Completion rates without a tenaculum were lower in nulliparous than in parous women (78% versus 86%). Average visual analog pain scores were lower with Carevix alone (35 ± 21) than when a tenaculum was required (43 ± 21). Most participants (73%) reported less pain than expected, and only 3% reported more pain. Among providers, 82% were satisfied with Carevix, citing ease of use, visibility, and low patient discomfort.

**Conclusions:**

In a multicenter real‐world setting, the novel, atraumatic device demonstrated feasibility and safety, with high completion rates and high satisfaction among both operators and patients.

## 1 INTRODUCTION

Intrauterine devices (IUD) represent a highly effective, long‐acting, reversible contraceptive method suitable for both parous and nulliparous women.^1^ Contraceptive failure in the first year of use is 0.8–1 per 100 woman‐years^2^ and user satisfaction is high.^3^, ^4^, ^5^ IUD releasing 52 mg levonorgestrel further provide endometrial protection during postmenopausal hormone therapy and are considered the first‐line treatment for heavy menstrual bleeding.^6^ Despite these advantages, IUD are underused, particularly among nulliparous women.^7^, ^8^

Pain or anticipated pain during device placement remains an important barrier to wider IUD use.^7^, ^8^, ^9^, ^10^ Pain experienced during IUD insertion is multifactorial, arising from procedural steps, such as the cervical tenaculum used to stabilize the cervix, uterine sounding and device placement.^11^, ^12^ Although multiple factors influence pain perception or reporting, including sociocultural factors, previous gynecologic examination, and a history of dysmenorrhea, insertion pain levels appear to be higher in nulliparous than in parous individuals, and the fear of possible pain is prominent in this group.^13^, ^14^, ^15^ In addition, some healthcare providers (HCP) underestimate patient pain.^16^ The US Centers for Disease Control and Prevention emphasize counseling on potential pain and different pain management options.^1^ In the UK, improving pain management during IUD procedures is a national healthcare priority.^17^

Alternative tools are being developed to reduce pain and cervical bleeding during insertion. An alternative is the Allis clamp, which is associated with less bleeding at the time of insertion,^18^ but which has not been conclusively shown to decrease procedural pain. The Carevix® cervical stabilizer (Aspivix SA, Epalinges, Switzerland) is an atraumatic device which uses suction to hold the cervix during IUD insertion.

An open‐label pilot study and a randomized, prospective, single‐blinded, interventional study have shown Carevix to be associated with high procedural completion rates and major significant reductions in pain during IUD placement compared with the standard single‐tooth tenaculum, including in nulliparous women.^19^, ^20^

These studies involved 113 participants at two centers. Controlled trials demonstrate the safety and efficacy of Carevix, but their findings may not fully reflect routine clinical practice. Real‐world data are therefore important to confirm its feasibility, usability, and generalizability. Our study aimed to evaluate procedural completion rates, pain, and bleeding during IUD placement with Carevix in everyday clinical practice across multiple centers, as well as the experiences of patients and operators.

## 2 MATERIALS AND METHODS

### 2.1 Design

This prospective, single‐arm, post‐marketing study evaluated user experiences with Carevix in an uncontrolled population. Data were collected from 19 sites in Austria, Brazil, France, Germany, Italy, Sweden, Switzerland, the UK, and the USA (see Appendix S1). Of 1250 procedures recorded between August 23, 2023, and January 6, 2025, 1178 were IUD procedures (insertion, removal, and replacement). In total, 55 procedures were excluded: 19 in contraindicated participants and 36 incomplete with Carevix or tenaculum. Hence, 1123 IUD procedures were included in the analysis. A non‐probabilistic convenience sampling method was employed.

All operators followed standard procedures at their centers. Participants were included at the discretion of the respective HCP. Each woman received the IUD of her choice, ensuring free selection. The exclusion criteria were defined in accordance with the manufacturer's Instruction for Use, reflecting the contraindications of Carevix for IUD procedures (cervix diameter smaller than 26 mm, severe vaginal bleeding, visible, symptomatic cervical or vaginal infections, cervical cancer, previous cervical surgery causing cervix deformation (e.g.conization/loop electro‐surgical excision), large ectropy, scar tissue, or any cervix abnormalities that do not permit a good adaptation of the device to the cervix).

The operators were staff physicians and residents from private practices or university hospitals, with no specific selection criteria. All invited operators consented to participate in the program and to receive training in device use either through on‐site training or via an online training program provided by the sponsor. Training included a presentation, reference to the Instructions for Use, and a quick guide to ensure correct handling. Devices were provided at no cost, and some operators received compensation for completing the questionnaire.

Use of analgesics (e.g. ibuprofen) or anesthesia (lidocaine spray, *n* = 9 hypnosis, *n* = 10 nitrous oxide inhalation, *n* = 2 larynx intubation, or larynxmask *n* = 2) were permitted. Operators could switch from Carevix to standard tenaculum if necessary. Data on HCP and participant experiences were collected using standardized questionnaires completed by the HCP and patients shortly after the procedure. The study protocol was approved by the ethical committees of the participating centers (Ethical committee of Campinas University and Scientific Review Committee of Indiana University, Indiana Clinical and Translational Sciences Institute), where required according to local regulations and the participants signed informed consent.

### 2.2 Study device

**FIGURE 1:**
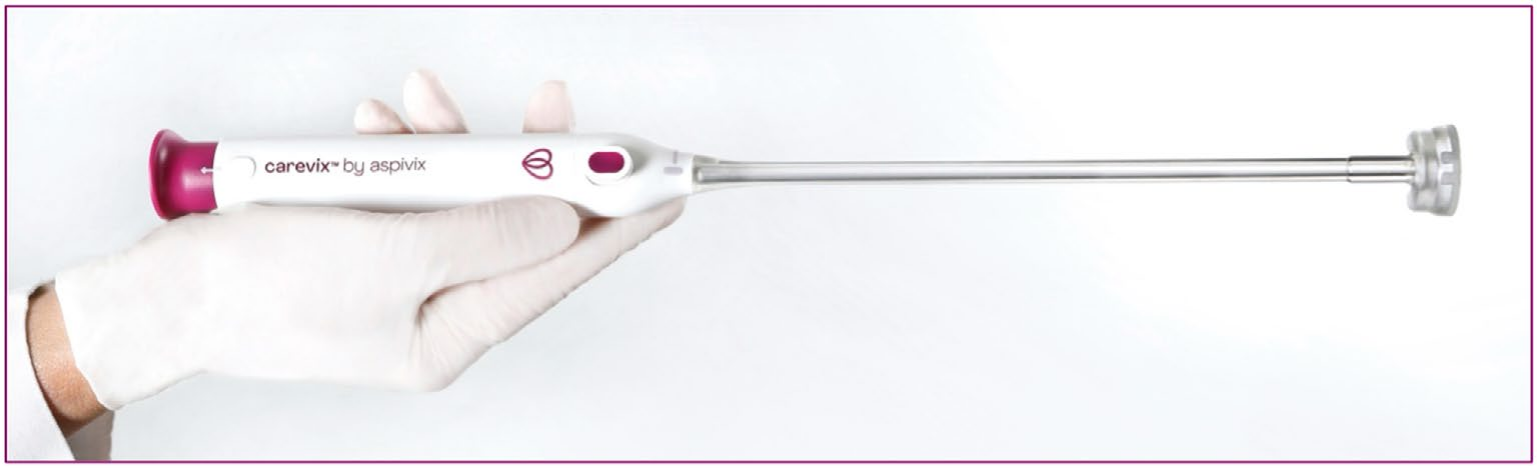
Carevix® suction device used for atraumatic cervical stabilization during transervical procedures.

The Carevix suction cervical stabilizer (Figure 1) is an atraumatic single‐use device. Its semi‐circular suction pad is designed to conform to the natural anatomy of the external cervical os, to gently grasp and hold the cervix during transcervical procedures. Tissue is released by simple release of the vacuum.

### 2.3 Analyzed variables

We recorded the number of IUD procedures performed using Carevix. The percentage of procedures completed using Carevix only (without the need for tenaculum assistance) was compared with the total number of procedures completed. Procedural results were analyzed for subgroups based on IUD insertion tube diameter, and for participants grouped according to uterus position, parity, and menstrual phase at placement.

Participant‐reported pain was measured on a 0‐ to 100‐mm Visual Analog Scale (VAS) using similar rulers accross centers. Pain scores were also analyzed for participants grouped according to age in 10‐year intervals and by analgesic and anesthetic use. Data on pain score, age, insertion tube diameter and IUD type were not available for all participants; therefore, analyses were performed on the subset of women with complete data.

The potential learning curve with Carevix was assessed by stratifying procedures according to HCP experience: 1–9 procedures, 10–19, and more than 19 procedures, respectively. For each group, we analyzed the completion rates using only Carevix, average pain scores, and HCP satisfaction. HCP reported their perceived time gained using Carevix, assessed relative to the increasing number of procedures using the same grouping. No multivariable adjustment was performed to separate operator learning from patient‐related factors. HCP and participant satisfaction were assessed on the number of individuals agreeing or disagreeing with specific statements after each procedure.

### 2.4 Safety

Operators were instructed to document any cases of bleeding that required management and report them. Centers were asked to report device defects or adverse events directly to Aspivix SA using its complaint form. For other safety data, the centers followed local reporting procedures.

### 2.5 Statistical analysis

Descriptive statistics (numbers/percentages, or median, range and interquartile range) summarized patient characteristics, procedural outcomes, and satisfaction ratings. Pain scores are presented as means ± standard deviations. Differences in pain scores across age groups were assessed using one‐way analysis of variance. Post‐hoc pairwise comparisons were conducted using Tukey honest significant difference test. A value of *P* of 0.05 or less was considered significant. Analyses were conducted using R version 4.3.0 (R Core Team, Vienna, Austria).

## 3 RESULTS

Table 1 summarizes the study cohort characteristics. Most participants were aged between 18 and 30 years, with less than 5% (42/992) aged below 18 years and 1% (3/992) over 50 years. Nearly half (46%, 513/1123) were nulliparous and a similar proportion (47%, 526/1123) were menstruating at the time of insertion. Seventy‐nine operators performed the procedures; only one had previous experience with the device. The number of procedures per operator ranged from 1 to 221 (median 5; interquartile range 2–13). Most IUD were levonorgestrel 52 mg‐releasing IUD. Analgesics were used in 196 procedures (17%) and anesthetics in 23 (2%); both types of agents were used in eight procedures (0.7%).

**TABLE 1:** Characteristics of the study cohort (total *n* = 1123).^a^

Variables	Value
Age, years (*n* = 992)	
<18	42 (4.2%)
18–30	559 (56.4%)
31–40	260 (26.2%)
41–50	128 (12.9%)
≥50	3 (0.3%)
Menstrual cycle status (*n* = 1123)	
Menstruating at the time of the procedure	526 (46.8%)
Parity (*n* = 1123)	
Nulliparous	513 (45.7%)
Parous	610 (54.3%)
Insertion tube diameter used in procedures, mm (*n* = 1098)	
>4	801 (72.9%)
<4	297 (27.1%)
IUD type (*n* = 1106)	
Hormonal IUD	857 (77.5%)
Copper IUD	249 (22.5%)
IUD used (*n* = 1112)	
Mirena, Kyleena, Jaydess	885 (79.6%)
MonaLisa/Multiload	89 (8.0%)
Optima (TCu380A)	80 (7.2%)
Gynefix	22 (2.0%)
CCD	17 (1.5%)
Other	19 (1.7%)

*Note:* CCD is a brand name for copper IUDs that include UT380 NT380 short and NT380 standard.

Abbreviation: IUD, intrauterine device.

^a^ Data are presented as number (percentage).

### 3.1 Procedural completion rates using Carevix

**FIGURE 2:**
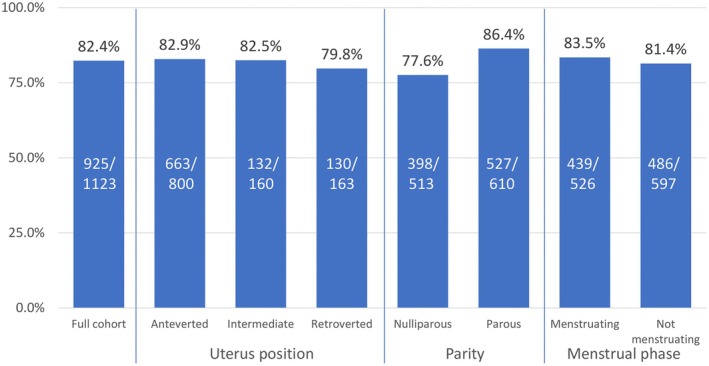
Number and percentages of procedures completed using Carevix only, according to uterus position, parity, and phase of menstrual cycle, respectively.

IUD procedures were completed using Carevix alone in 925/1123 participants (82%). Tenaculum was additionally used in 198 procedures (18%), most commonly due to vacuum loss (*n* = 194, 98%). Uterine position or phase of the menstrual cycle did not influence the need for additional tenaculum use (Figure 2). Although tenaculum use was more frequent in nulliparous women, over three‐quarters of insertions in this group (398/513) were completed using Carevix alone. Procedures using insertion tubes with diameter smaller than 4 mm had a slightly higher completion rate with Carevix alone (*n* = 257/297; 86%) than those using inserter tubes with diameter of 4 mm or larger (*n* = 652/801; 81%).

### 3.2 Participant‐reported pain

**FIGURE 3:**
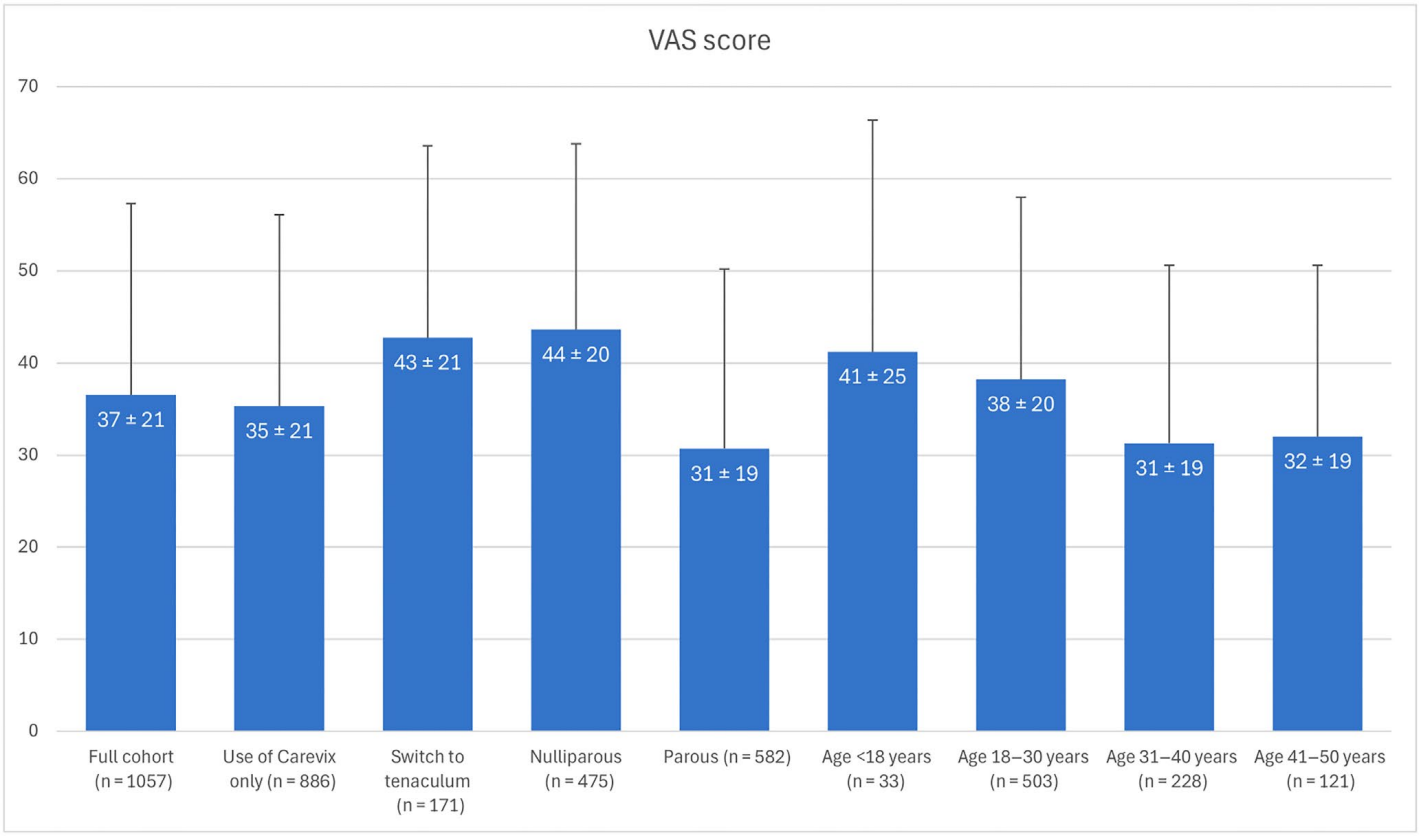
Participant‐reported pain scores in the IUD cohort and subgroups.

Due to missing data, analyses were conducted only on those with complete pain score information. Average VAS scores in the full IUD cohort (1057/1123) were 37 ± 21. Average pain scores were significantly lower in procedures with Carevix only (35 ± 21) than in those that needed tenaculum assistance (43 ± 21) (*P* < 0.001), though this difference was not clinically relevant. Parous women reported lower pain scores (31 ± 19) than nulliparous participants (44 ± 20) (*P* < 0.001). Age data were available for 992 participants (88%), of whom 885 had pain scores recorded. Post‐hoc comparisons revealed that participants aged 31–40 years reported significantly lower pain scores than those aged 18–30 years (*P* < 0.001), whereas all other pairwise comparisons were not statistically significant (*P* > 0.05) (Figure 3). Menstrual status (menstruating: 37 ± 20 versus non‐menstruating: 36 ± 21) and IUD type (hormonal: 37 ± 21 versus copper: 36 ± 21) did not significantly affect pain scores (*P* > 0.05).

Average VAS pain scores were 39 ± 24, 38 ± 22, and 37 ± 20 for operators with 1–9 (298 procedures), 10–19 (136 procedures), and more than 19 (689 procedures) IUD insertions. Use of analgesic was associated with the highest pain scores (44 ± 22; *n* = 188) versus (36 ± 20; *n* = 910) (*P* < 0.001) without the use of anesthesia or analgesic.

Procedures with wider insertion tubes had lower pain scores (36 ± 20; *n* = 768) than those with insertion tubes less than 4 mm in diameter (41 ± 23; *n* = 265) (*P* = 0.002).

### 3.3 Safety

Cervical bleeding requiring management by caregivers was recorded for 38 procedures (3.4%). Of these cases, 13 of 161 (8.1%) were associated with the use of tenaculum, whereas 25 of 926 (2.6%) used Carevix alone. In 26 cases of bleeding (67%) a spontaneous device release was observed. Bleeding was minimal and typically resolved rapidly using a compress; within 1 minute in 32 procedures (82%) and within 2 minutes in six procedures (18%). No vasovagal reactions (fainting, syncope, or spasms) or adverse events, and no device defects were reported during this study.

### 3.4 Patient and HCP satisfaction

**FIGURE 4:**
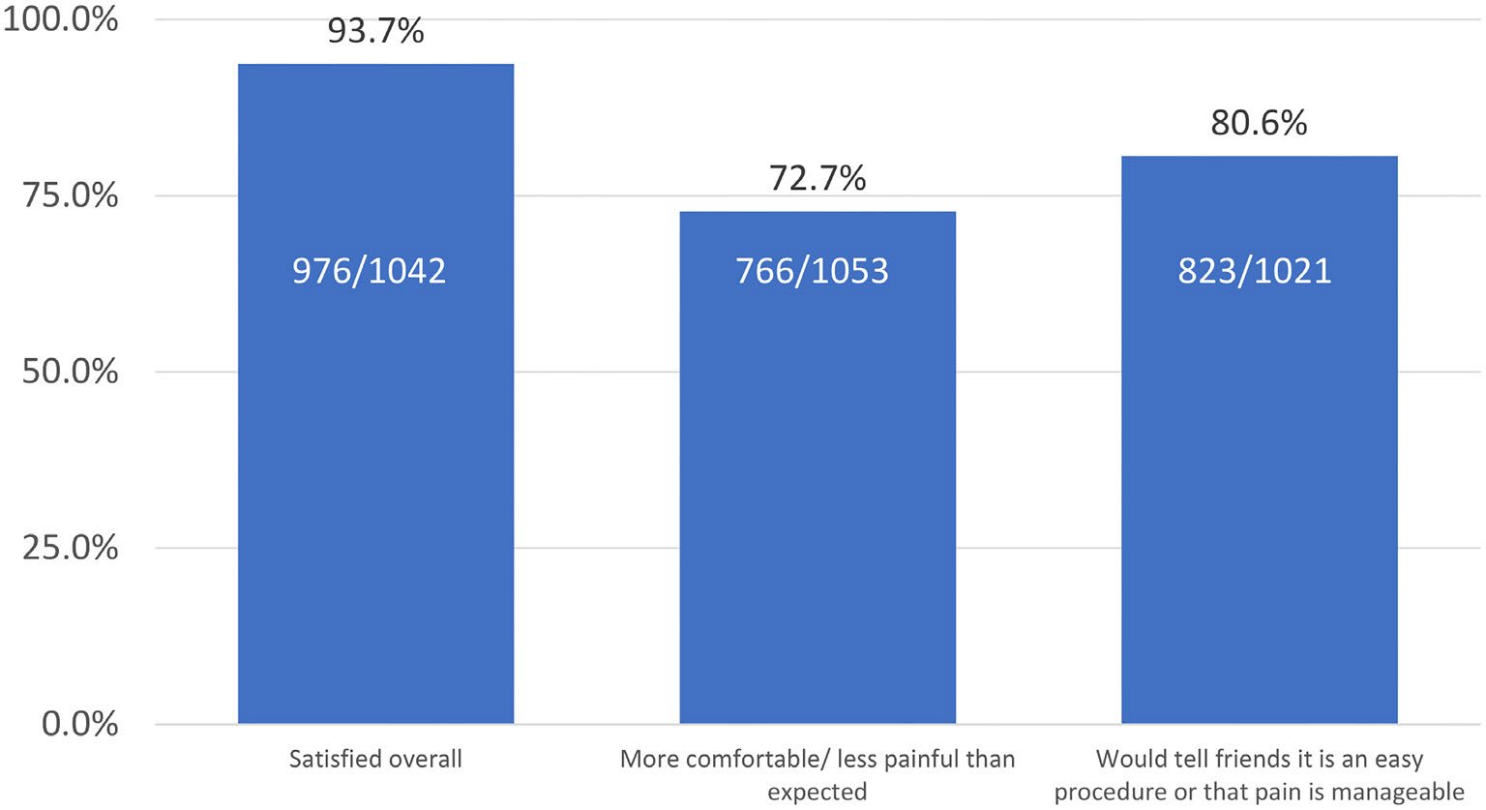
Percentages of participants agreeing strongly with statements on satisfaction with the IUD procedures.

Patient satisfaction was high (Figure 4), with only 2% (25/1042) reporting dissatisfaction. Most participants (766/1053; 73%) considered it less painful than expected and only 32 (3%) reported “much more” pain than expected. Most participants (823/1021; 81%) would tell friends that the procedure was easy, and that pain was manageable; an additional 191 (19%) would recommend it with the reservation that some pain should be expected.

**FIGURE 5:**
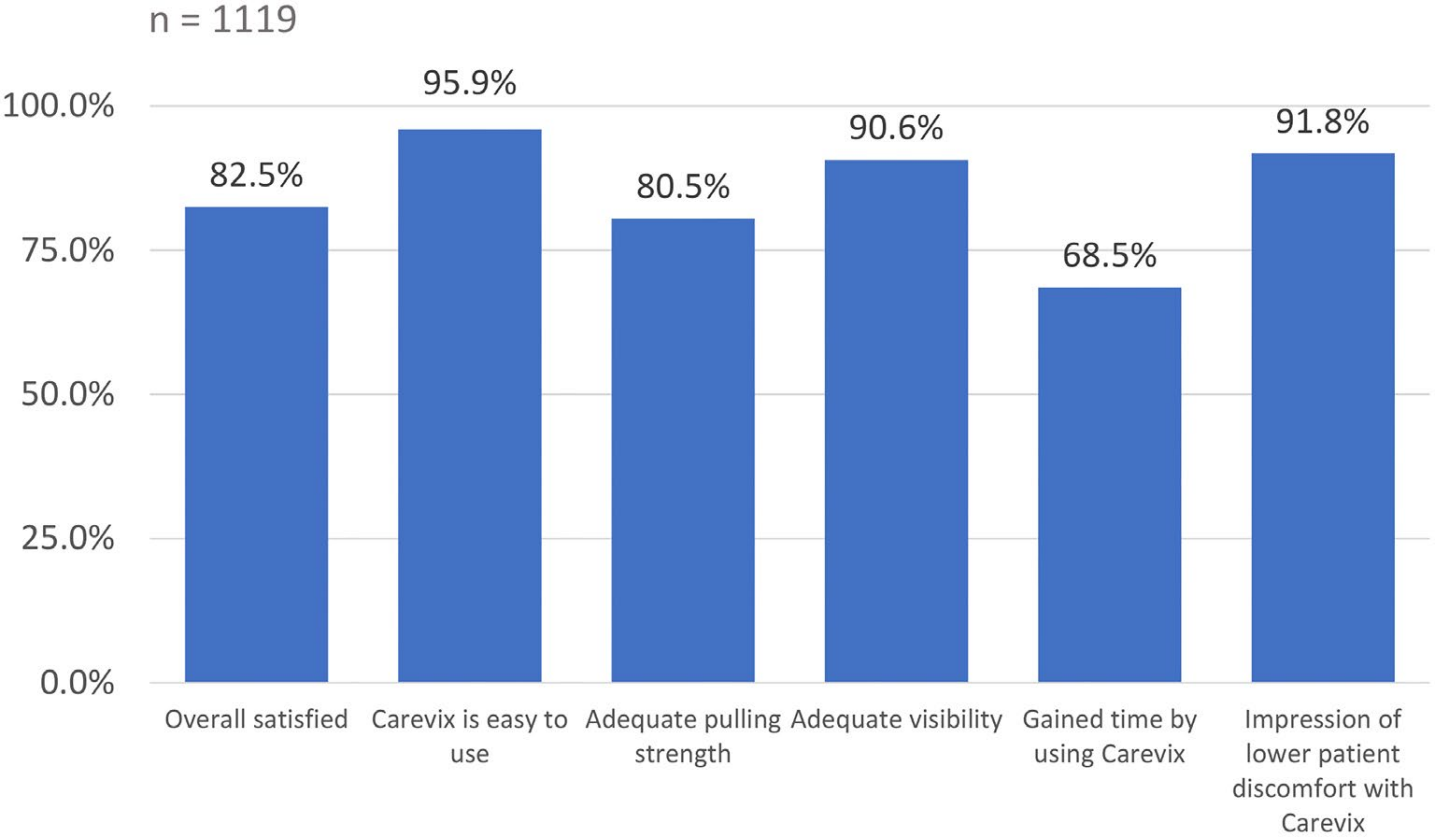
HCP satisfaction scores. Numbers refer to procedures for which operators “agreed” or “agreed strongly” with the statements provided.

Among HCP, 82% (923/1119) were overall satisfied using Carevix (Figure 5). Ease of use, adequate visibility, and a perception of low patient discomfort were the most favorable characteristics, reported in at least 90% of procedures. Operators' first nine procedures were completed using Carevix only in 237 (80%) cases, increasing to 84% after the first 19 procedures. After 1–9 procedures, 52% of operators felt that they gained time with Carevix versus tenaculum; after 19 or more procedures, this percentage had risen to 78%.

## 4 DISCUSSION

Our results of 1123 IUD placements showed that 82% were completed without tenaculum use, indicating suitability for diverse anatomies and participant characteristics.

Our data support previous findings that reported an 83% successful insertion rate with Carevix, comparable to the 82% observed in our study.^20^ The average observed VAS pain scores were slightly higher than in the randomized study^20^ (VAS score 31.5 at the IUD placement versus 35 of our study). These scores fall below the predefined typical pain threshold for IUD placement, which ranges from a VAS of 40 to 61.^10^ Neither menstruation status nor uterus position affected the outcomes or the application of Carevix, indicating no major risk of vacuum loss at certain insertion angles. Inserter diameter appeared to influence completion rates with Carevix alone and pain scores, with larger diameters associated with more frequent need for tenaculum but lower pain scores. This is in contrast to a previous study with mostly nulliparous adolescents, which reported higher pain scores in IUD insertion procedures with larger inserter diameter.^21^ This difference may reflect variations in study populations and the multifactorial nature of pain during IUD placement. Our analysis focused specifically on pain during the IUD placement step.

The least painful procedures tended to be those performed without the use of anesthetic or analgesic. Although intra/para‐cervical block with lidocaine are mostly recommended,^1^, ^22^ those were rarely used (*n* = 9, 0.8%) in our sample. Nonetheless, women who request anesthetic or analgesic may have lower pain tolerance or higher anxiety regarding the procedure. Consequently, their reported pain scores may not be representative of the entire sample, and values from this subgroup could act as a potential confounder.

Differences in pain scores between Carevix and tenaculum procedures may reflect the atraumatic design of the study device or the need to switch to a tenaculum. The device's vacuum‐assisted grasping mechanism offers a gentler alternative to the single‐tooth tenaculum, thereby reducing tissue trauma and discomfort.

The atraumatic nature of Carevix was associated with less bleeding requiring management (2.6%), a lower rate compared with tenaculum use,^23^ consistent with previous studies.^19^, ^20^ In this study, pain scores were compared between procedures performed with Carevix alone and those in which a tenaculum was additionally used. As tenaculum use for cervical stabilization is one of the sources of pain and bleeding, atraumatic alternatives may reduce barriers to IUD use. As the present cohort represents multiple centers, operators, and treatment conditions, we believe the recorded pain scores approximate typical use, though generalizability is limited by selection, measurement, and reporting biases.

Satisfaction with IUD procedures is inversely related to experiences of pain.^24^ We found high satisfaction scores, similar to previous reports with Carevix.^19^, ^20^ Three out of four participants reported the procedure to be less painful or “more comfortable” than expected. Operators varied in their procedural experience and professional backgrounds. Considering that only one HCP had experience with the device, it is plausible that the additional tenaculum use was influenced by the operator's limited familiarity or confidence with the device rather than by an inherent limitation of the device itself. Nevertheless, satisfaction rates with the Carevix device were high even from the first 1–9 procedures, indicating a straightforward familiarization process.

Key strengths of this study include the large sample size, multicenter setting, focus on routine practice, and inclusion of multiple IUD models. Limitations include the incomplete data, lack of comparator group and potential courtesy bias from face‐to‐face questionnaires. Pain scores, bleeding rates, and HCP/participant satisfaction can only be inferred by comparisons with other studies and could be speculative. Furthermore, the underrepresentation of adolescents (<18 years old: 4.2%) and older women (>50 years old: 0.3%), along with the predominance of hormonal IUD limits the generalizability. Subgroup differences, such as parity, were not explored. Women's adherence to Carevix remains uncertain, particularly if future use involves out‐of‐pocket costs or routine clinical implementation. Nevertheless, Carevix's benefits in reducing pain^20^ and enhancing patient comfort and operator satisfaction suggest its potential value for routine use. This study was funded by the manufacturer, which may pose bias.

In conclusion, the atraumatic Carevix device used for cervical stabilization at IUD placement in a multicenter global real‐life setting, showed high procedural completion rates, low pain and bleeding rates, and high satisfaction scores among both HCP and participants.

## AUTHOR CONTRIBUTIONS

MY and LB contributed to conceptualization, data curation, formal analysis, supervision, validation, writing—original draft, and writing—review and editing; VC contributed to data curation, supervision, and writing—review and editing; and DM, ACM, XEA, EG, JB, EGV, MM, CTJ, and AC contributed to data curation, investigation, and writing—review and editing.

## FUNDING INFORMATION

The study was sponsored by ASPIVIX SA. The authors had full control over the study protocol, conduct, analysis and conclusions, and the decision to publish.

## CONFLICT OF INTEREST STATEMENT

MY declares advisory board participation for Exeltis Suisse SA. LB and CJ have participated in advisory boards at Bayer. The other authors have no conflicts of interest.

## Supporting information


**Appendix S1.** List of centers and patients enrolled.

## Data Availability

The data sets are available from the corresponding author on reasonable request.
