# Supplementary material for: Safety and efficacy of a suction cervical stabilizer for IUD insertion: Results from a multicenter post‐marketing study
Source: Int J Gynaecol Obstet. 2025 Dec 3;173(2):1067–74. doi: 10.1002/ijgo.70718 (PMC13094678; doi:10.1002/ijgo.70718)
Supplement: Supplementary file 1 — Appendix S1. List of centers and patients enrolled. [file IJGO-173-1067-s001.docx]

## Appendix

### List of centers and patients enrolled:

University of Campinas, Brazil: 668 procedures, 5 operators

Hôpitaux Universitaires de Genève (Gynécologie), Switzerland: 210 procedures, 20 operators

Capio Barnmorskemottagning Malmö Centrum, Sweden: 72 procedures, 5 operators

La Clinique des Femmes, France : 56 procedures, 3 operators

Indiana University Health, US: 41 procedures, 12 operators

Femme Frauenarztpraxis, Germany: 28 procedures, 1 operator

Hôpitaux Universitaires de Genève (Urgences), Switzerland: 26 procedures, 9 operators

Praxis Centrogyn, Germany: 26 procedures, 1 operator

Individual contributors, Switzerland: 26 procedures, 1 operator

Peterborough City Hospital, Great Britain: 19 procedures, 7 operators

Centre de santé sexuelle Bienne, Switzerland: 14 procedures, 6 operators

Kantonsspital St. Gallen, Switzerland: 13 procedures, 1 operator

Frauenarztzentrum Penzberg, Germany: 13 procedures, 1 operator

Ganzheitliche Frauenarztpraxis, Germany: 11 procedures, 1 operator

Medizinisches Zentrum Margareten, Austria: 10 procedures, 1 operator

Villa Donatello, Italy: 7 procedures, 1 operator

MediZentrum Schüpfen AG, Switzerland: 5 procedures, 2 operators

Evangelisches Klinikum Köln Weyertal, Switzerland: 3 procedures, 1 operator

Centre hospitalier universitaire vaudois (CHUV), Switzerland: 2 procedures, 1 operator
